# Multi-modality imaging findings of huge intrachoroidal cavitation and myopic peripapillary sinkhole

**DOI:** 10.1186/s12886-018-0681-x

**Published:** 2018-02-02

**Authors:** Yutong Chen, Xiaoli Ma, Rui Hua

**Affiliations:** Department of Ophthalmology, First Hospital of China Medical University, No. 155 Nanjingbei Street, Heping District, Shenyang, Liaoning Province People’s Republic of China

**Keywords:** *En face* technique, Intrachoroidal cavitation, Multi-color, Myopic peripapillary sinkhole

## Abstract

**Background:**

Peripapillary intrachoroidal cavitation was described as the presence of an asymptomatic, well-circumscribed, yellow-orange, peripapillary lesion at the inferior border of the myopic conus in eyes with high myopia.

**Case presentation:**

A 66-year-old myopic Chinese man was enrolled and his multi-color imaging examination showed a well-circumscribed, caesious, peripapillary lesion coalesced with the optic nerve head vertically rotated and obliquely tilted, together with an inferotemporal sinkhole in the myopic conus. The optical coherence tomography images showed an intrachoroidal hyporeflective space, schisis, an intracavitary septum located below the retinal pigment epithelium and inserted beneath the optic nerve head, as well as a sinkhole between the peripapillary intrachoroidal cavitation and the vitreous space.

**Conclusions:**

Both myopic colobomas and sinkhole in myopic conus may contribute the coalescence of intrachoroidal cavitation with optic nerve head. These qualitative and quantitative new findings will be beneficial for understanding its pathomorphological mechanism, and the impact on optic nerve tissue of myopic patients.

## Background

Peripapillary intrachoroidal cavitation was first described by Freund et al. [1] using optical coherence tomography (OCT). However, the presence of a yellow-orange peripapillary lesion on OCT is not always indicative of intrachoroidal schisis or cavitation [2]. Toranzo et al. [3] re-evaluated these lesions and observed an intrachoroidal hyporeflective space with normal overlying retinal pigment epithelium (RPE) and retina. The gradual sinking of peripapillary retinal tissue into a sclerochoroidal cavity associated with retinal hole formation and posterior vitreous prolapse was termed “myopic peripapillary sinkhole” [4].

## Case Presenctation

**Fig. 1:**
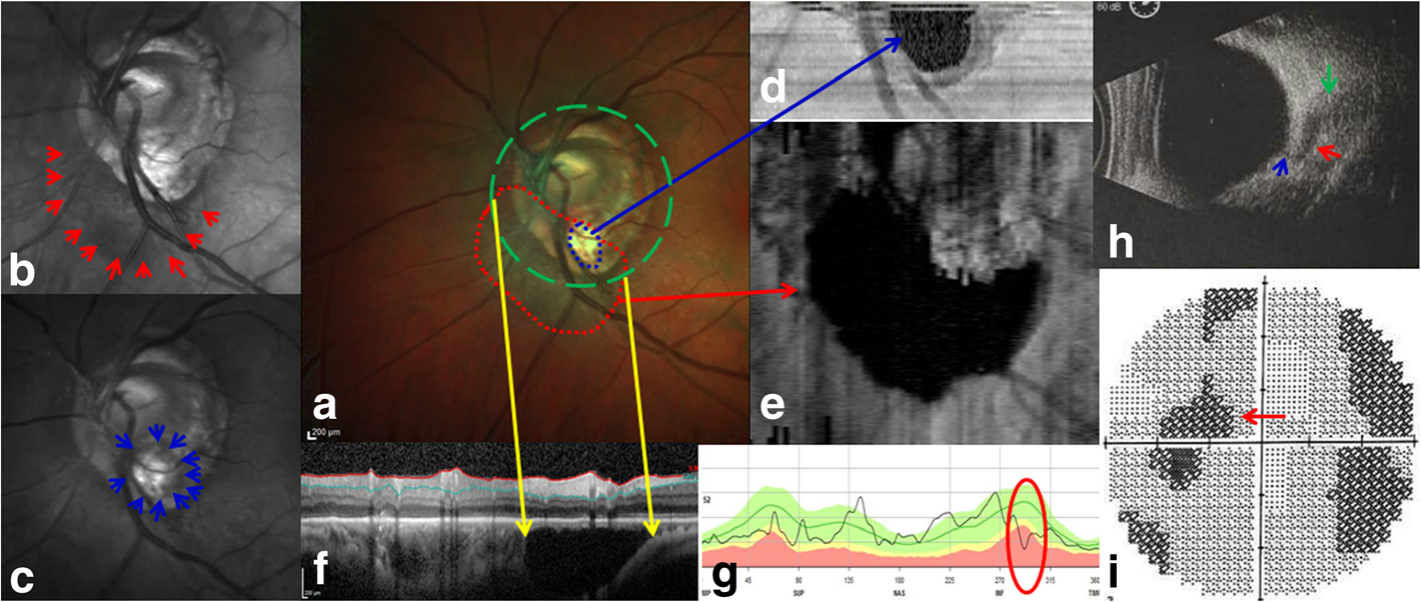
Identification of peripapillary intrachoroidal cavitation and sinkhole on multi-modality images. **a** Multi-color images of peripapillary intrachoroidal cavitation and tilted ONH show a well-circumscribed, caesious, peripapillary lesion coalesced with the ONH (red dots) and its inferotemporal sinkhole (blue dots) in the myopic conus. The inferotemporal retinal vein covers the sinkhole of the peripapillary intrachoroidal cavitation in the myopic conus. **b** Infrared reflectance demonstrating a peripapillary dark reflective region (red arrows), corresponding to the red dots in (**a, c**). A clear border of the sinkhole (blue arrows) could be observed in green reflectance, corresponding to the blue dots in (**a, d**). The sinkhole (size: 0.34 mm^2^) on an *en face* image (blue arrow). **e** The intrachoroidal cavity (size: 7.85 mm^2^) on an *en face* image (red arrow). **f** EDI-OCT (green dashed circle in **a**) showing the entire profile of the peripapillary intrachoroidal cavitation (yellow arrows). **g** A thinner retinal nerve fiber was detected above the peripapillary intrachoroidal cavitation region (red circle), in accordance with the EDI-OCT image in (**f, h**). The coalescence of peripapillary intrachoroidal cavitation (red arrow) and ONH (green arrow) as evident on an ocular B scan ultrasound image, as well as the sinkhole (blue arrow), consistent with (**a**). **i** Visual field testing shows that the dark defects (red arrow) connected with the blind spot, consistent with the peripapillary intrachoroidal cavitation location in (**a**) and (**h**). (ONH, optic nerve head; EDI-OCT, enhanced depth imaging optical coherence tomography)

**Fig. 2:**
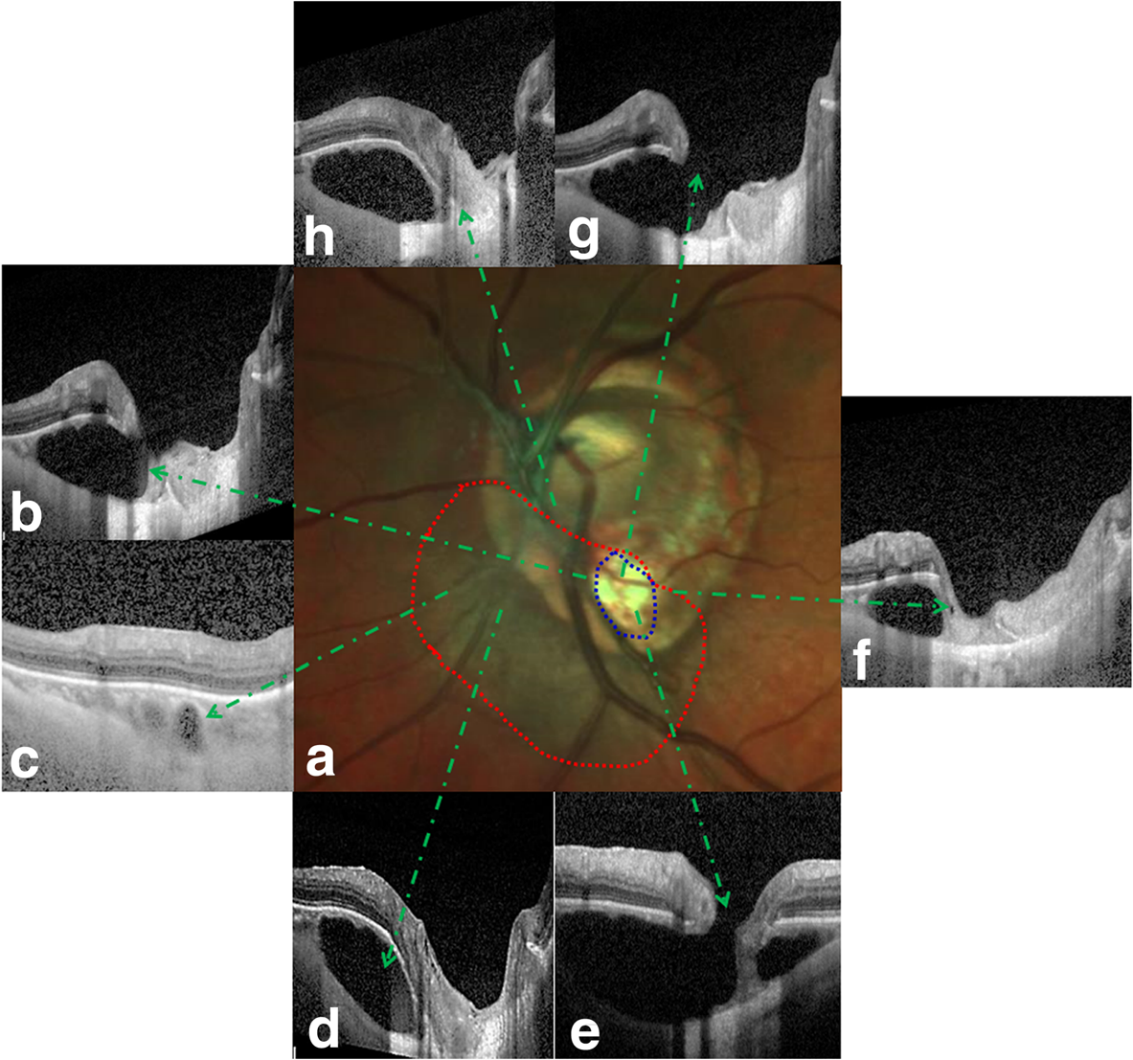
The OCT profiles of the peripapillary intrachoroidal cavitation and sinkhole. **a** The location of the peripapillary intrachoroidal cavitation (red dots), sinkhole (blue dots), and tilted ONH. **b** The small cleft in the retinal tissue on the nasal side of the sinkhole, with the discontinued RPE and deformed anterior lamina cribosa. **c** The small intrachoroidal cavity on the nasal border of the peripapillary intrachoroidal cavitation. **d** The entire peripapillary intrachoroidal cavitation inserted beneath the ONH. **e** The intracavitary septum was observed near the sinkhole. **f** The RPE was discontinuous and the inner retina was collapsed temporal to the sinkhole also with the deformed anterior lamina cribosa. **g** A complete view of the sinkhole, peripapillary intrachoroidal cavitation, discontinuity of the retinal layers over the pit-like defect with herniation of retinal tissue through which communication between the choroidal cavity and subretinal space was seen. **h** The peripapillary intrachoroidal cavitation coalesced with the ONH with the deformed anterior lamina cribosa. (ONH, optic nerve head; OCT, optical coherence tomography; RPE, retinal pigment epithelium)

Herein, we reported the new findings of this lesion based on multimodality imaging qualitatively and quantitatively. A 66-year-old myopic Chinese man was referred to our clinic and multi-color imaging showed a well-circumscribed, caesious, peripapillary lesion coalesced with the optic nerve head (ONH) vertically rotated and obliquely tilted, together with an inferotemporal sinkhole in the myopic conus (Fig. 1). The corresponding changes were also evident on ocular B scan ultrasonography images, and visual field testing, showing an enlargement of the blind spot and a superior arcuate scotoma in the left eye (Fig. 1). The size of the peripapillary intrachoroidal cavitation and sinkhole could be measured on *en face* images, and was found to be 7.85 mm^2^ and 0.34 mm^2^, respectively (Fig. 1). The enhanced depth imaging (EDI) OCT images showed an intrachoroidal hyporeflective space, schisis, an intracavitary septum located below the RPE and inserted beneath the ONH, as well as a cleft (sinkhole) between the peripapillary intrachoroidal cavitation (presumably liquid vitreous humor) and the vitreous space (Fig. 2). This study adhered to the tenets of the Declaration of Helsinki, and the Medical Research Ethics Committee of China Medical University approved this study. Consent form was obtained from the patient.

## Discussion and conclusions

One hypothesis regarding the pathogenesis of peripapillary intrachoroidal cavitation involves the scleral extension around the ONH [5]. Comparable morphologic changes observed at the border of colobomas and peripapillary intrachoroidal cavitation on OCT are different stages of the same disorder [6]. Because of the presence of a cleft-like communication between the retina and the choroid with vitreous prolapse and anomalous vessels, some investigators believe that the anomaly is congenital in origin [7]. Toranzo et al. [3, 8] presumed that the progression of the staphyloma breaks the collagenous limiting tissue of Elschnig between the choroid and the optic nerve, resulting in a retraction of the choroid away from the optic nerve margin.

However, peripapillary intrachoroidal cavitations do not occur exclusively in highly myopic eyes [2]. It is believed that there is a true break because repeated scans demonstrated the existence of an intact conus-lesion junctional membrane immediately adjacent to the break [9]. The myopic conus and surrounding peripapillary tissue may be structurally weaker, and with aging, there may be a decrease in the amount of absorbing fluids originating from the vitreous cavity. Due to malnutrition arising from the absence of the choroid in the conus, the roof of the cavern (retinal nerve fiber layer) gradually collapses [2]. Additionally, the progression of peripapillary staphyloma may stretch and disrupt the tissue at the edge of the myopic conus, and the adherence of the retina and the RPE at the conal margin prevents the break from opening to the subretinal space. Instead, vitreous fluid gradually gains access into the choroidal tissue, creating schisis or a fluid pocket [9].

Sometimes, a myopic sinkhole is formed to facilitate the posterior flow of vitreous into the sclerochoroidal hollow, causing peripapillary choroidal thickening and cavitation [4]. Moreover, Cadherin or another cell adhesion molecule may possibly play an important role in the formation of peripapillary intrachoroidal cavitations [6].

Finally, there are several differences and advantages in our case, compared with Ohno-Matsui K’s study [10]. We reported the relationship between peripapillary intrachoroidal cavitation and sinkhole, which were detected by the combination of multicolor imaging and EDI-OCT. Multicolor has advantages over fundus color photograph in detecting “pits” in Ohno-Matsui K’s study [10]. Additionally, we compared the findings of OCT and multicolor images with the ocular B scan ultrasonography images and visual field testing results, which were new for Ohno-Matsui K’s study. Differently, the sinkhole in our study, seem to be circular, and the inferotemporal retinal vein covered the sinkhole of the peripapillary intrachoroidal cavitation in the myopic conus.

Both myopic colobomas and sinkhole in myopic conus may contribute the coalescence of intrachoroidal cavitation with optic nerve head. We hope that these qualitative and quantitative new findings will be beneficial for understanding its pathomorphological mechanism, and the impact on optic nerve tissue.

## Abbreviations

OCT: Optical coherence tomography
ONH: Optic nerve head
RPE: Retinal pigment epithelium
